# On the use of Different Methodologies in Cognitive Neuropsychology: Drink Deep and from Several Sources

**DOI:** 10.1080/02643294.2012.672406

**Published:** 2012-07-03

**Authors:** Lyndsey Nickels, David Howard, Wendy Best

**Affiliations:** 1 ARC Centre of Research Excellence in Cognition and its Disorders (CCD), Macquarie University, Sydney, Australia; 2 School of Education, Communication and Language Sciences, Newcastle University, Newcastle upon Tyne, UK; 3 Division of Psychology and Language Sciences, University College London, London, UK

**Keywords:** Case series, Single-case studies, Cognitive neuropsychology, Methodology

## Abstract

Cognitive neuropsychology has championed the use of single-case research design. Recently, however, case series designs that employ multiple single cases have been increasingly utilized to address theoretical issues using data from neuropsychological populations. In this paper, we examine these methodologies, focusing on a number of points in particular. First we discuss the use of dissociations and associations, often thought of as a defining feature of cognitive neuropsychology, and argue that they are better viewed as part of a spectrum of methods that aim to explain and predict behaviour. We also raise issues regarding case series design in particular, arguing that selection of an appropriate sample, including controlling degree of homogeneity, is critical and constrains the theoretical claims that can be made on the basis of the data. We discuss the possible interpretation of “outliers” in a case series, suggesting that while they may reflect “noise” caused by variability in performance due to factors that are not of relevance to the theoretical claims, they may also reflect the presence of patterns that are critical to test, refine, and potentially falsify our theories. The role of case series in treatment research is also raised, in light of the fact that, despite their status as gold standard, randomized controlled trials cannot provide answers to many crucial theoretical and clinical questions. Finally, we stress the importance of converging evidence: We propose that it is conclusions informed by multiple sources of evidence that are likely to best inform theory and stand the test of time.

In the development of theories of cognitive processes, cognitive neuropsychology has been unusual within cognitive psychology in promoting the use of single-case studies (e.g., Caramazza, 1986). In addition, many researchers within cognitive neuropsychology have vehemently argued against the use of group studies where data are averaged across cases (e.g., Caramazza & McCloskey, 1988; Sokol, McCloskey, Cohen, & Aliminosa, 1991). Nevertheless, it has always been the case that research articles would often include more than a single case (e.g., Warrington, 1975). These cases are examined for similarities (associations) and differences (dissociations) in the patterns of performance that are used to provide further evidence in the development of the theory. More recently, the practice of including multiple cases has become more common, and data are often presented from multiple single cases (e.g., Goldrick, Folk, & Rapp, 2010). These case series investigations also share the aim of informing cognitive theories, enabling us to explain and predict the behaviour of the unimpaired and impaired cognitive system.

Traditionally, theoretical accounts in cognitive neuropsychology have been on the basis that a general cognitive theory is being tested with data from brain-damaged patients on the assumption that the theory constrains the possible forms of impairment. At a more sophisticated level, a theory might require certain relationships between symptoms. So, for instance, the Dell and O'Seaghdha (1991) model predicted only a certain set of possible rates of particular error types; any error rates outside this set would provide evidence against the adequacy of the model. Hence, any person with impaired word retrieval who produces an error pattern outside these limits is a challenge to, and possible disproof of, the model. This kind of evidence requires just a single patient to challenge the adequacy of the theory. Nickels and Howard (1995b) explored lesions to Dell and O'Seaghdha's (1991) model and pointed out that there should be a positive correlation between the proportion of semantic errors and the proportion of phonological errors that are real words. This prediction of the model cannot be tested with a single case. Nickels and Howard's (1995b) claim relates to the relationship between different kinds of errors that should be observed across a case series. Indeed, Nickels and Howard found that the relationship did not hold.

Hence, the point we wish to make at the outset of this discussion is that a theoretical account can be tested both with data from single subjects and from case series; neither has privileged value, but both are important. We therefore wholeheartedly agree with Schwartz and Dell (2010) when they argue that case series methodology provides a useful complement to single-case studies in cognitive neuropsychological and cognitive neuroscience research: Single-case and case series methodologies each bring unique strengths to the field. We have been publishing case series of people with aphasia for more than 25 years, in relation to both their patterns of performance and their response to intervention (e.g., Best, Herbert, Hickin, Osborne, & Howard, 2002; Best, Hickin, Herbert, Howard, & Osborne, 2000; Howard, Patterson, Franklin, Morton, & Orchard-Lisle, 1984; Nickels & Howard, 1994, 1995a, 1995b, 1996). Like Schwartz and Dell, we too have argued for the relevance of the importance of case series (e.g., Howard, 2003). However, we differ from Schwartz and Dell in our view of what constitutes a case series. We believe that a case series is indeed a concatenation of multiple single-case studies with constraints noted by Schwartz and Dell: For this concatenation to be a “true” case series, it should include data on the same tasks and materials. It is also the case that (by necessity) the cognitive analysis may often be more restricted than for single-case reports. Schwartz and Dell argue that case series design primarily aims to examine covariance across measures of cognitive performance and understand the cognitive mechanisms underlying this covariance. While this is a potential aim, we feel that the term “case series design” can be applied more widely, with case series reports of this type representing a subset of the larger set of case series used to inform theoretical debate.

## Dissociations and associations

Both associations and dissociations in performance are interesting and important in contributing to theory development: Here, we agree, once again, with Schwartz and Dell (2010). Critically, both dissociations and associations can be examined using single-case and case series methodology. Nevertheless, in the literature there has been a tendency for single-case studies to be linked to the methodology of dissociations and case series to associations (e.g., Patterson & Plaut, 2009). We do not dispute that cognitive neuropsychology may have paid less attention to associations than dissociations in the past. Certainly, descriptions of cognitive neuropsychological logic have often focused on the importance of the “double dissociation” in single-case studies (e.g., Coltheart, 2002; Shallice, 1988). While clearly a powerful methodology, in fact single cases have also been used to inform theoretical models using methods other than the double dissociation. Schwartz and Dell provide the example of Warrington (1975) who provided associations between performance on words and pictures and a dissociation with other nonsemantic language tasks to demonstrate the profile of semantic memory impairment. Hence, a cornerstone of the single-case approach is the use of converging evidence for associations and dissociations across tasks. However, critically, the single-case approach goes beyond measures of accuracy to examine error types and qualitative differences between individuals. For example, it is not merely that M.F. (Best, 1996) is poor at picture naming that is of interest. It is also the fact that he produces formal paraphasias (errors that are real words that share phonemes with the target, e.g., a CART named as “card”) that is used as support for models of language production in which there is feedback from phonological to lexical representations (Goldrick, 2006). Similarly it is not that M.K. (Howard & Franklin, 1988) makes errors in spoken picture naming that is critical for informing theoretical accounts, but that he produces “orthographic” errors. For example, when naming a picture of an irregularly spelled word, such as a picture of a BEAR, M.K. may produce “beer”—a regularized pronunciation of the target: It is the occurrence of these errors that led Howard and Franklin to propose a “graphemic-to-orthographic” conversion route that converts graphemes (activated via the orthographic output lexicon) to input orthographic codes (which can then be read aloud using the sublexical grapheme–phoneme conversion route). These are data that depend on a single (unusual) case, but, we want to argue, they are relevant for the understanding of the cognitive architecture of language processing in people without language impairment. That depends on appreciating that single cases are important and should not be discounted (as “outliers” or “exceptions”); those that use correlational methods must believe in the reliability of the differences between their subjects and, therefore, cannot lightly dismiss data from single subjects as exceptions. Correlational analyses necessarily require (some degree of) confidence in the differences in the levels of performance of participants in the constituent tasks—people interested in associations and those interested in dissociations will both, necessarily, be committed to believing that their data reveal real differences between participants (although subject to statistical noise). The consequence is that case series can yield data about both associations and dissociations.

This is not to say that every apparent dissociating case is a true dissociation. There are situations where it is possible that an apparent dissociation results from, for example, the use of an alternative strategy. Blind acceptance of dissociating cases is no better than blind rejection. Data on these cases must be robust and replicable to exclude the possibility of alternative strategies or noisy data (e.g., from “bad days”). Nevertheless, we would argue that even a single “true” dissociating case is important when evaluating associations. We illustrate this using the example cited by Schwartz and Dell (2010) of the phonological deficit hypothesis for phonological dyslexia.

Patterson and Plaut (2009) report an association in patients with acquired phonological dyslexia, who are poor at reading nonwords. Such individuals are poor at producing and/or judging information about the sounds of written letter strings, and the typical association is with deficits in phonological tasks that do not involve written-word stimuli (e.g., segmentation and blending tasks). Patterson and Plaut (2009) therefore argued that a phonological impairment was the cause of phonological dyslexia. In a test of this hypothesis, Crisp and Lambon Ralph (2006) found a correlation between nonword reading accuracy and nonreading phonological skills in a group of individuals with nonword reading impairments. Their conclusion is that “phonological”^1^ impairments (in the absence of semantic impairments) result in phonological dyslexia, with specifically, the extent of the nonword reading impairment being linked to the extent of the “phonological” impairment.

Howard and Nickels (2005) report two cases, M.M.G. and H.B., both of whom show impairments in nonword repetition, digit span, and phonological manipulation tasks (e.g., segmenting the first sound from a spoken nonword). Thus these two individuals have the defining features of phonological impairment described by Crisp and Lambon Ralph (2006). The prediction from the phonological deficit account of acquired phonological dyslexia is that H.B. and M.M.G. should be phonological dyslexics with impaired nonword reading. M.M.G. does indeed show this pattern, but H.B. shows nonword reading performance that is within normal limits for accuracy and latency. This is despite the fact that his ability to perform phonological tasks is as severely impaired as that of M.M.G. and many other phonological dyslexics (e.g., Patterson& Marcel, 1992). Hence, H.B.'s dissociating pattern provides clear evidence against the hypothesis that acquired “phonological” impairments necessarily cause nonword reading impairments.^2^ Indeed, Howard and Nickels (2005) suggest that the association could have a causal relationship in the reverse direction. Unimpaired subjects use orthography to support their performance in tasks requiring “phonological” manipulation, while those participants with phonological dyslexia and poor nonword reading will have a reduced support. Hence impaired orthographic skills may result in impairment on phonological manipulation task: the reverse pattern to that claimed by the phonological deficit hypothesis. In summary, it remains the case that a single dissociating case can challenge a hypothesis made on the basis of an association and should not be cast aside as an exception.

A parallel situation arises in the anatomical case series that are discussed by Schwartz and Dell (2010). They describe data from Schwartz et al. (2009) who found brain regions associated with semantic errors in naming when controlling for comprehension: the left anterior temporal lobe and particularly the left mid to anterior middle temporal gyrus. They claim that damage to this area is associated with postsemantic, prephonological generation of semantic errors. However, once again, if there is just one individual with postsemantic errors who does not have a lesion in the anterior temporal lobe (or wherever may be claimed) then such a lesion is not necessary for postsemantic errors. And, conversely, if there is one individual without postsemantic errors who has a lesion in this area then it is not sufficient for these errors. That is, just as in behavioural studies concerned with cognitive theories, anatomical studies, concerned with localization of cognitive processes, also require converging evidence from both associations and dissociations, and the latter can call into question conclusions from the former.

## Sampling and heterogeneity in case series

A further important issue in case series investigations is sampling. This is particularly pertinent when associations are the tool used to evaluate theoretical accounts. To illustrate our point, we return to the work of Crisp and Lambon Ralph (2006) who chose individuals with symptoms of phonological or deep dyslexia (defined by a lexicality effect, image-ability effect, or semantic errors in reading aloud). Given that their primary aim was to examine the characteristics of phonological/deep dyslexia, this was an entirely appropriate sample. However, as discussed above, Crisp and Lambon Ralph also used the data to argue that the cause of the reading impairment is the underlying phonological (and semantic) impairment. They state, for example, “The data from this study suggest that poor nonword reading is directly related to the underlying phonological impairment,” (p.359) and “Assessments of phonological and semantic impairments suggested that the integrity of these primary systems underpinned the patients’ reading performance” (p. 348). However, in order to make these claims, a rather different sample is required: a sample of individuals who are chosen because they are impaired in semantics and/or phonology regardless of the characteristics of their reading. Such a sample may show different patterns to that of a sample of individuals with phonological/deep dyslexia. Moreover, such a sample could include individuals, like H.B. (Howard & Nickels, 2005) described above, whose pattern of performance calls into question the hypothesis being addressed.

It is clear that choosing an appropriate sample is not as straightforward as it might at first appear. Great care must be taken in ensuring appropriate sampling for case series, and the conclusions drawn on the basis of case series data should be limited to those that the sample can truly support. Researchers need to define their theoretical question and determine the characteristics of the sample needed to investigate this question. The importance of sampling brings us to a related issue, that of heterogeneity in case series and its control.

### Heterogeneity

Schwartz and Dell (2010) suggest that one of the important differences between group designs and case series designs is in the treatment of heterogeneity. Heterogeneity is avoided in group studies where it is viewed as a problem: The mean of a group is taken to be a good estimate of the mean of that population from which that group is drawn; any deviations from the mean for the individual subjects are taken to be measurement error or individual differences without interest (just variation about the mean). In contrast, in case series, Schwartz and Dell argue that heterogeneity is vital, and that it is through exploration of the causes of heterogeneity that case series can advance theory. However, it is important to consider exactly what we mean here by heterogeneity and what the underlying assumptions might be. In particular it is important to draw the distinction between heterogeneity in the sense of variability in the dependent variable and heterogeneity meaning variability in other aspects of performance such as level of impairment.

We agree that variability in the dependent variable is essential: In order to perform meaningful correlations, it is important that there be variability in the factor of interest. For example, if every individual had a naming accuracy of 30%, it would be impossible to investigate which factors affected the success of word retrieval across the case series.^3^ Nevertheless, other forms of heterogeneity in the dependent variable may not be as desirable. For example, naming accuracy may be affected by attentional factors or visual processing impairments. This would result in variability in the dependent variable, but for reasons that would not inform our understanding of word retrieval from the mental lexicon. Therefore, there needs to be both variability in performance and homogeneity in the source of the variability in the dependent variable: To achieve this homogeneity, participants need to be excluded who have impairments that influence the dependent variable but are not related to the cognitive process under consideration.

In a similar way, there needs to be homogeneity in other characteristics of the sample. The issue is how homogeneous does the sample need to be and on which measures? There is no easy answer to this question, as which factors should be controlled will vary with the theory and research questions, but it is critical that the inclusion and exclusion criteria for a sample be both clear and theoretically motivated. For example, if the aim of an investigation is to examine the factors affecting the production of semantic errors in word production, what exclusion criteria might be appropriate? In a strictly feed-forward theory of word production, phonological processing will have no impact on the likelihood of semantic error production. Hence, it may be thought that individuals who produce semantic errors but also have phonological impairments could be sensibly excluded. However, in a highly interactive theory where activation at later stages of processing feeds back to earlier stages of processing, it is possible that impairments in phonological processing could have an impact on semantic error production. Therefore, in order to discriminate between these theories, both sets of individuals need to be included, and analyses need to be carried out to compare them. However, even if the focus is on testing a feed-forward theory alone (perhaps in the absence of an alternative theory), there is an argument for including individuals who produce semantic errors and have phonological impairments: It is possible that phonological processes will be found to have an impact on the likelihood of semantic error production, which, in turn, would refute the feed-forward theory.

### Summary: Sampling and heterogeneity in case series investigations

Sampling is a complex issue in case series and one that perhaps has not received the attention it deserves in the literature. The participants selected will constrain the theoretical claims that can be made; particular care must be taken to ensure claims do not go beyond those that are warranted by the sample.

When selecting participants for case series investigation, it is important that:

- The case series should have a clear theoretical motivation.
- The hypotheses to be tested are used to constrain the suitable participants (and in turn the theoretical claims that are able to be supported by the data).
- There is no evidence of input impairments at a prior level of processing that would affect critical measures (e.g., unrelated visual input impairments when investigating reading).
- There is no evidence of output impairments below the level of processing being investigated that would affect critical measures (e.g., motor speech impairments when investigating word retrieval).
- Performance is above floor and below ceiling to enable sensitivity to changes in the variable under consideration.
- When case series are used to test a causal relationship, sampling includes individuals on the basis of each associated impairment independently.

## Heterogeneity and the treatment of outliers

In their discussion of heterogeneity, Schwartz and Dell (2010) note that when case series analysis is used to test a model, each case can be characterized as consistent with the model or not. In other words, the data can be examined and outliers detected. This raises the question of how one is to determine what is an outlier.

It is important that statistics be brought to bear in the examination of whether individuals in a case series are showing equivalent effects. For example, Best et al. (2002) examined the effects of phonological and orthographic cues on word retrieval. A homogeneity test (Leach, 1979) was used to demonstrate that there were differences across the individuals in the case series in the effectiveness of cues on word retrieval. In contrast, using the same test, Howard, Hickin, Redmond, Clark, and Best (2006) demonstrated that there was no significant difference in the effects of word–picture matching on subsequent naming across the individuals in their case series. This was despite performance at posttest (where pretest was zero) varying from 13% to 88% correct. Similarly, Best (2005) found no significant difference in the therapy effect for children included in a case series intervention study, suggesting that, despite the children's different language and learning profiles, the intervention was working in the same way.

Assuming that outliers are demonstrated to differ statistically from the rest of the cases, then they may be of two types: An outlier may be a real example of a dissociating pattern that can falsify a theory. For example, Castles, Bates, and Coltheart (2006) examined reading performance of a large sample of children unselected for reading ability (2,066 developing readers and 1,026 older readers). Most children showed similar performance on reading of irregular words and nonwords as would be expected given the high correlation found between these two abilities. However, a small proportion (10–11%) showed a pattern of developmental surface dyslexia, where nonword reading was within normal limits (less than half a standard deviation below the mean), and irregular word reading was severely impaired (more than 1.64 standard deviations below the mean). In addition, there were outliers in the other direction, developmental phonological dyslexics, where nonword reading was poor, and irregular word reading was unimpaired. Castles et al. then used these outliers as evidence against a connectionist model of reading acquisition (Harm & Seidenberg, 1999), which is unable to simulate pure cases of surface and phonological dyslexia.

An alternative to “real” outliers occurs when outliers are due to an additional confounding factor. Take, for example, a hypothetical study that examined the relationship between word retrieval and semantic processing as measured by picture naming and spoken word–picture matching respectively. If an individual had an auditory processing impairment, this would “artificially” lower the individual's score on word–picture matching such that it no longer representeda“true” measure of their semantic processing. Consequently, this individual may appear as an outlier in terms of the general trend for poorer picture naming to be correlated with poorer word–picture matching. Schwartz and Dell (2010) propose that explanations for outliers can be sought through additional assessment. We agree that the explanation could be found by additional assessments but disagree strongly that such assessments should be reserved for those who deviate from the trends. Such a fishing expedition runs the risk of providing a post hoc explanation that may prove to be false when all tasks are carried out by all participants. For example, as a result of additional testing, one may claim that participant X doesn't accord with our hypothesis because she or he has characteristic Y. However, it may turn out that one or some or many of the participants who accord with the hypothesis also show characteristic Y. Hence, any additional testing must be performed with all of the individuals in the case series in order for such a claim to be made. We would advocate that it is vital that case series analyses be combined with full cognitive characterization.

## Case series in investigations of the effects of treatment

One important area that was not covered by Schwartz and Dell (2010) in their consideration of case series as a methodology was the use of case series in intervention studies. Despite the fact that randomized controlled trials (RCTs) are the gold standard for treatment efficacy, it remains a fact that RCTs, just like any group study, involve averaging across participants, and hence a positive result means that “on average” a treatment is effective. It is often the case that the treatment is not effective for every individual, or that the overall size of the effect is very small (but significant through the size of the group). The problems with RCTs in the field of aphasia have long been acknowledged (e.g., Hegde, 2007; Howard, 1986), and single-cases/case series designs (e.g., Perdices & Tate, 2009; Tate et al., 2008; Thompson, 2006) are widely used to evaluate the efficacy of treatment at the level of the individual. More recently, these designs (labelled as *n* of 1 randomized controlled trials) have begun to become more widespread even in medicine (e.g., Guyatt et al., 1990). However, once again, there are limitations to what the single-case study design can tell us regarding treatment, and replication across a series of single cases is recognized as important. It is only through case series that we can determine which factors are important in the efficacy of treatment, by, once again, searching for associations and dissociations between the dependent variable (here response to treatment) and other measures (e.g., those reflecting the nature of the impairment). For example, as described above, Best et al. (2002) examined the effects of phonological and orthographic cues as facilitators of word retrieval across a case series of people with aphasia. They analysed the effects of cueing for each participant individually and then used the data from all the individuals to investigate the mechanisms underlying the effectiveness of the cues. Through a series of correlations, they determined that severity of the naming impairment did not relate to effectiveness, but that success of orthographic cueing was predicted by accuracy of nonword reading (specifically the accuracy of the first letter or phoneme). In contrast, success of phonemic cues did not correlate with accuracy of nonword repetition. On the basis of these data, Best et al. were able to draw conclusions regarding the mechanisms by which orthographic and phonological cues were operating and constrain theories of language production.

Hence, both clinically and theoretically, treatment studies can benefit from case series methodology with the same constraints as those outlined above for studies not involving treatment (see for further discussion, Howard, 2000, 2003).

## Converging evidence from different designs

As we noted above, Schwartz and Dell (2010) argue that case series are important as an alternative and complement to single-case study design. We would like to go further and argue that single cases and case series both play an essential part of theory building. We would argue against a slow drift away from the depth and rigour that a well designed and executed single-case study can bring to theoretical debate: In case series, inevitably, the reported data are less complete. The advantage of good single-case studies is that multiple converging sources of evidence from different experimental paradigms can be brought to bear on a single theoretical issue. While, in principle, this is possible in case series, it does not happen in practice (for good practical reasons that include time, participant compliance, etc.).

It is our view that consideration of data from multiple experimental designs (including case series and single cases; treatment and nontreatment) and from multiple sources (both intact and impaired populations; developmental and acquired disorders; treatment studies and nontreatment) is most likely to inform theories in such a way that they will stand the test of time. A recent example is the burgeoning interest in phonological neighbours as predicting the ease of lexical retrieval. Neighbourhood density has been shown to be important in typically developing children (Storkel & Lee, 2011), in skilled adult readers (Grainger, Muneaux, Farioli, & Ziegler, 2005), and in children with language impairments (German & Newman, 2004). These are all group studies. Our primary interest is in the value that can be added by detailed investigation with single-case and case series studies. Here we find better performance in aphasic naming for items with more phonological neighbours in a single case (e.g., Best, 1995) and that several aspects of overlap including initial segment contribute independently to the production of (nonsemantic) word errors in a case series (Goldrick et al., 2010). These findings with strong implications for models of typical and impaired language production result from careful investigation of qualitative as well as quantitative aspects of performance. Similarly, Graham and Hodges (1997) provide a rare example of the use of converging evidence from different designs—both group study and single-case investigation to inform our understanding of how different brain areas are employed in retaining memories over time. Just as in the example focusing on neighbourhood size, this demonstrates the benefits of combining across research designs.

In addition, it is important that evidence be combined across populations. All too frequently, researchers focus on one population: unimpaired adults, typically developing children, language-impaired adults, or language-impaired children. The research in each of these fields is often guilty of appearing unaware (or even being unaware) of that carried out in the other fields. Indeed, even within research involving the same populations this can happen. For example, research on literacy development and literacy disorders deriving from psychology and from education take very different perspectives that could benefit from dialogue. Similarly, even within cognitive psychology, research on short-term memory is rarely combined with research on language perception and production (but see Jacquemot & Scott, 2006, for an exception).

In sum, converging evidence has always been a cornerstone of cognitive neuropsychology in order to determine the level of impairment. However, the use of converging evidence should be broader, converging across designs and populations. The volume edited by Brenda Rapp (2001) *“The Handbook of Cognitive Neuropsychology”* is an excellent example of this approach. Each of the chapters explicitly brings together relevant evidence not just from cognitive impairments (usually single cases), but also from cognitive psychology (usually groups), computational modelling, and neuroscience, neatly demonstrating how these sources of evidence combine to produce a whole greater than the sum of the contributing parts.

## Conclusion

In conclusion, we agree with many but not all of the points about case series raised by Schwartz and Dell (2010), and we welcome the opportunity to explore these issues. As Hegde (2007) notes, “researchers select experimental designs based on their training, experience, expertise, and research philosophy. And it will continue to be that way. Those who typically use a particular strategy will retain a healthy critical disposition toward the one they do not use. This is good for the science … because the skeptics of any approach will help keep the enthusiasts a notch below extremists” (p. 30).

We have discussed issues relating to dissociations and associations, which we believe are too often thought of as the primary tools of cognitive neuropsychology. In fact, the identification of associations and dissociations are simply two, of a number, of methods that aim to explain and predict behaviour. We have argued that, for case series, selection of an appropriate sample, including controlling type of heterogeneity, is critical and constrains the theoretical claims that can be made on the basis of the data. We note that outliers in a case series may indeed be “noise” caused by variability in performance due to factors that are not of relevance to the theoretical claims. Alternatively, however, these outliers may be those critical, rare cases that cognitive neuropsychology has long embraced in the testing and development of cognitive theories. It is vital that, as case series become more frequent, these “true” outliers retain the status they deserve. We have also discussed the role of case series in treatment research and note that, despite their status as gold standard, randomized controlled trials cannot provide answers to many crucial theoretical and clinical questions. Indeed, Kaptchuk (2001) makes the observation “while a gold standard is valuable, any worshiping at an altar of a golden calf, like the Biblical Exodus story, may obscure ‘reality’… . Unless one is aware of a research methodology's weaknesses, scientific activity may become a mechanical ritual” (p. 548).

The point we should like to focus on is the importance of converging evidence: We propose that conclusions based on clearly articulated theory and informed by multiple sources of evidence are likely to best inform theory and stand the test of time. We have also argued that one cannot afford to dismiss findings that conflict with a theoretical position as inconvenient or misleading data. The title for Patterson and Plaut's (2009) paper was a quotation from Alexander Pope's (1711) poem “A Little Learning”. We adapt the first two lines of this poem (with our addition in brackets):

A little learning is a dang'rous thing, drink deep [and from several sources], or taste not the Pierian spring”^4^

(p. 14).

## Footnotes

1 We refer to “phonological” in quotation marks, in order to indicate that this refers to phonology as indexed by the ability to perform tasks involving phonological manipulation (e.g., segmentation, blending) and not necessarily to, for example, a phonological impairment that results in phonological errors in word production.

2 We acknowledge the situation may be different in development where poor phonological skills are a strong predictor of dyslexia in longitudinal studies (Muter, Hulme, Snowling, & Stevenson, 2004). In this field, there are several reports of cases with normal phonological processing and poor reading (often developmental surface dyslexia, e.g., Castles & Coltheart, 1996) and normal readers of nonalphabetic languages with poor phonological manipulation skills (e.g., Read, Yun-Fei, Hong-Yin, & Bao-Qing, 1986).

3 However, it would still be possible to examine which factors affected success of naming items within each individual and compare this across the case series.

4 In Greek mythology, the Pierian Spring of Macedonia was sacred to the Muses, as the metaphorical source of knowledge of art and science.

## References

[R1] Best W. (1995). A reverse length effect in dysphasic naming: When elephant is easier than ant. Cortex.

[R2] Best W. (1996). When racquets are baskets but baskets are biscuits, where do the words come from?: A single case study of formal paraphasic errors in aphasia. Cognitive Neuropsychology.

[R3] Best W. (2005). Evaluation of a new intervention for word-finding difficulties in children. International Journal of Language and Communication Disorders.

[R4] Best W., Herbert R., Hickin J., Osborne F., Howard D. (2002). Phonological and orthographic facilitation of word retrieval in aphasia: Immediate and delayed effects. Aphasiology.

[R5] Best W., Hickin J., Herbert R., Howard D., Osborne F. (2000). Phonological facilitation of aphasic naming and predicting the outcome of treatment for anomia. Brain and Language.

[R6] Caramazza A. (1986). On drawing inferences about the structure of normal cognitive systems from the analysis of patterns of impaired performance: The case for single-patient studies. Brain and Cognition.

[R7] Caramazza A., McCloskey M. (1988). The case for single-patient studies. Cognitive Neuropsychology.

[R8] Castles A., Bates T., Coltheart M. (2006). John Marshall and the developmental dyslexias. Aphasiology.

[R9] Castles A., Coltheart M. (1996). Cognitive correlates of developmental surface dyslexia: A single case study. Cognitive Neuropsychology.

[R10] Coltheart M., Wixted J., Pashler H. (2002). Cognitive neuropsychology. Stevens’ handbook of experimental psychology, third edition: Vol. 4. Methodology.

[R11] Crisp J., Lambon Ralph M. A. (2006). Unlocking the nature of the phonological–deep dyslexia continuum: The keys to reading aloud are in phonology and semantics. Journal of Cognitive Neuroscience.

[R12] Dell G. S., O'Seaghdha P. G. (1991). Mediated and convergent lexical priming in language production: A comment on Levelt et al (1991). Psychological Review.

[R13] German D. J., Newman R. S. (2004). The impact of lexical factors on children's word-finding errors. Journal of Speech, Language, and Hearing Research.

[R14] Goldrick M. (2006). Limited interaction in speech production: Chronometric, speech error, and neuropsychological evidence. Language and Cognitive Processes.

[R15] Goldrick M., Folk J., Rapp B. (2010). Mrs. Malaprop's neighborhood: Using word errors to reveal neighborhood structure. Journal of Memory and Language.

[R16] Graham K. S., Hodges J. R. (1997). Differentiating the roles of the hippocampus complex and the neocortex in long-term memory storage: Evidence from the study of semantic dementia and Alzheimer's disease. Neuropsychology.

[R17] Grainger J., Muneaux M., Farioli F., Ziegler J. (2005). Effects of phonological and orthographic neighbourhood density interact in visual word recognition. Quarterly Journal of Experimental Psychology.

[R18] Guyatt G. H., Keller J. L., Jaeschke R., Rosenbloom D., Adachi J. D., Newhouse M. T. (1990). The n-of-1 randomized controlled trial: Clinical usefulness. Our three-year experience. Annals of Internal Medicine.

[R19] Harm M. W., Seidenberg M. S. (1999). Phonology, reading acquisition, and dyslexia, insights from connectionist models. Psychological Review.

[R20] Hegde M. N. (2007). A methodological review of randomized clinical trials. Communicative Disorders Review.

[R21] Howard D. (1986). Beyond randomised controlled trials: The case for case studies of the effects of treatment in aphasia. British Journal of Disorders of Communication.

[R22] Howard D., Papathanasiou I. (2000). Cognitive neuropsychology and aphasia therapy: The case of word retrieval. Acquired neurogenic communication disorders: A clinical perspective.

[R23] Howard D., Papathanasiou I., De Bleser R. (2003). Single cases, group studies and case series in aphasia therapy. The sciences of aphasia: From therapy to theory.

[R24] Howard D., Franklin S. (1988). Missing the meaning? A cognitive neuropsychological study of the processing of words by an aphasic patient.

[R25] Howard D., Hickin J., Redmond T., Clark P., Best W. (2006). Re-visiting “semantic facilitation” of word retrieval for people with aphasia: Facilitation yes but semantic no. Cortex.

[R26] Howard D., Nickels L. A. (2005). Separating input and output phonology: Semantic, phonological and orthographic effects in short-term memory impairment. Cognitive Neuropsychology.

[R27] Howard D., Patterson K. E., Franklin S. E., Morton J., Orchard-Lisle V. M., Rose F. C. (1984). Variability and consistency in picture naming by aphasic patients. Advances in neurology: Vol. 42. Progress in physiology.

[R28] Jacquemot C., Scott S. K. (2006). What is the relationship between phonological short-term memory and speech processing?. Trends in Cognitive Sciences.

[R29] Kaptchuk T. (2001). The double-blind, randomized placebo controlled trial: Gold standard or golden calf?. Journal of Clinical Epidemiology.

[R30] Leach C. (1979). Introduction to statistics: A non-parametric approach for the social sciences.

[R31] Muter V., Hulme C., Snowling M. J., Stevenson J. (2004). Developmental Psychology.

[R32] Nickels L. A., Howard D. (1994). A frequent occurrence—Factors affecting the production of semantic errors in aphasic naming. Cognitive Neuropsychology.

[R33] Nickels L. A., Howard D. (1995a). Aphasic naming—What matters?. Neuropsychologia.

[R34] Nickels L. A., Howard D. (1995b). Phonological errors in aphasic naming—Comprehension, monitoring and lexicality. Cortex.

[R35] Nickels L. A., Howard D. (1996). Missions in syllable deduction: Lexical stress effects in aphasia. Brain and Language.

[R36] Patterson K., Marcel A., Alegria J., Holender D., Juncade Morais J., Radeau M. (1992). Phonological ALEXIA or PHONOLOGICAL alexia?. Analytic approaches to human cognition.

[R37] Patterson K., Plaut D. (2009). “Shallow draughts intoxicate the brain”: Lessons from cognitive science for cognitive neuropsychology. Topics in Cognitive Science.

[R38] Perdices M., Tate R. (2009). Single-subject designs as a tool for evidence-based clinical practice: Are they unrecognised and undervalued?. Neuropsychological Rehabilitation.

[R39] Pope A. (1711). An essay on criticism.

[R40] Rapp B. (2001). The handbook of cognitive neuropsychology.

[R41] Read C., Yun-Fei Z., Hong-Yin N., Bao-Qing D. (1986). The ability to manipulate speech sounds depends on knowing alphabetic writing. Cognition.

[R42] Schwartz M. F., Dell G. S. (2010). Case series investigations in cognitive neuropsychology. Cognitive Neuropsychology.

[R43] Schwartz M. F., Kimberg D. Y., Walker G. M., Faseyitan O., Brecher A., Dell G. S. (2009). Anterior temporal involvement in semantic word retrieval: VLSM evidence from aphasia. Brain.

[R44] Shallice T. (1988). From neuropsychology to mental structure.

[R45] Sokol S. M., McCloskey M., Cohen N. J., Aliminosa D. (1991). Cognitive representations and processes in arithmetic: Inferences from the performance of brain-damaged subjects. Journal of Experimental Psychology: Learning, Memory & Cognition.

[R46] Storkel H. L., Lee S.-Y. (2011). The independent effects of phonotactic probability and neighbourhood density on lexical acquisition by preschool children. Language and Cognitive Processes.

[R47] Tate R., McDonald S., Perdices M., Togher L., Schultz R., Savage S. (2008). Rating the methodological quality of single-subject designs and n-of-1 trials: Introducing the single-case experimental design (SCED) scale. Neuropsychological Rehabilitation.

[R48] Thompson C. K. (2006). Single subject controlled experiments in aphasia: The science and the state of the science. Journal of Communication Disorders.

[R49] Warrington E. K. (1975). The selective impairment of semantic memory. Quarterly Journal of Experimental Psychology.

